# Flexible spectral manipulation property of a high power linearly polarized random fiber laser

**DOI:** 10.1038/s41598-018-20664-y

**Published:** 2018-02-01

**Authors:** Jun Ye, Jiangming Xu, Jiaxin Song, Hanshuo Wu, Hanwei Zhang, Jian Wu, Pu Zhou

**Affiliations:** 1 College of Optoelectronic Science and Engineering, National University of Defense Technology, Changsha, 410073 China; 2 Hunan Provincial Collaborative Innovation Center of High Power Fiber Laser, Changsha, 410073 China

## Abstract

Fiber lasers with flexible spectral manipulation property could provide a flexible tool for scenes where the temporal coherence property accounts, for example, coherent sensing/communication and nonlinear frequency conversion. Due to the good laser performance and relative simplicity of implementation, random fiber lasers (RFLs) based on random distributed feedback and Raman gain have earned more and more attention in the past few years, and a variety of RFLs with substantially different spectral properties have been developed. In this presentation, we demonstrate a high power linearly polarized RFL with flexible spectral manipulation property, in which the central wavelength and the linewidth of the spectrum can be tuned independently through a bandwidth-adjustable tunable optical filter (BA-TOF). The central wavelength of the RFL can be continuously tuned from 1095 to 1115 nm, while the full width at half-maximum (FWHM) linewidth has a maximal tuning range from ~0.6 to more than 2 nm. Moreover, the output power of 1102.5–1112.5 nm reaches ~23 W with polarization extinction ratio (PER) value > 20 dB. To the best of our knowledge, this is the first demonstration of a powerful linearly polarized RFL with both wavelength and linewidth tunability.

## Introduction

In 2010, Turitsyn *et al*. demonstrated a new type of random fiber laser (RFL), which is quite different from conventional fiber laser with well-defined resonator^1^. RFL makes use of Rayleigh scattering (RS) to produce random distributed feedback (RDFB) and stimulated Raman scattering (SRS) to provide amplification, demonstrating the most obvious features of cavity-free and mode-less^2^. Over recent years, RFLs have drawn a great deal of attention, and gradually lead to the realization of high power^3,4^, narrow-linewidth^5–7^, multi-wavelength^8,9^ and linearly polarized operation^7,10,11^. Thanks to the good laser performance and relative simplicity of implementation, RFLs have attracted a large variety of applications, such as frequency doubling to the visible range^12^, sensing and telecommunication^13–15^, pump source in mid-infrared laser and supercontinuum light source^16–19^, and stable seed for high power fiber master oscillator power amplifier (MOPA)^20,21^.

For better understanding the physical mechanism of random lasing and widening the application range in optical communication, sensing, secure transmission and other fields, various kinds of RFL schemes with wavelength tunability have been demonstrated^22–28^. The tunable operation of RFL is produced either with a wavelength-fixed pump and an optical filter or directly with a tunable pump source. Due to the relatively broad and flat profile of Raman gain, Babin *et al*. demonstrated a 1535–1570 nm tunable RFL by inserting a tunable filter into the cavity^22^. With an optical grating filter, Sarmani *et al*. achieved a 1550–1571 nm tunable RFL^23^. With a fiber Fabry-Perot cavity connected with a Mach-Zehnder interferometer, Zhu *et al*. reported a multi-wavelength RFL with tuning range from 1553.9 to 1565.4 nm^24^. To further widen the wavelength tuning range, reduce the lasing threshold and pursue more potential in power scaling, tunable RFLs using rare-earth-doped fiber as gain medium instead of Raman gain have been demonstrated^25,26^. Wang *et al*. proposed a tunable Er-doped RFL with a tunable fiber Fabry-Perot interferometer, which realized a broad tuning range up to 40 nm with a pump threshold as low as 13 mW^25^. With a piece of Yb-doped fiber and a manual tunable filter, Du *et al*. first reported a tunable RFL working at 1 μm, which can be tuned continuously from 1040 to 1090 nm^26^. However, the above reported tunable RFLs either exploit the gain spectrum of a single Raman shift or employ the rare earth ions only. Moreover, the operating bandwidths of the tunable optical filters are limited. So that the maximal tuning range is confined to a few tens of nanometers in these schemes. A combination of tunable pump source and cascaded SRS process could be a good candidate to achieve widely tunable RFLs. In this way, Zhang *et al*. demonstrated a RFL with an ultra-broadband wavelength tuning range of 300 nm^27^, even 900 nm^28^.

Although a variety of RFLs with linearly polarized output, wavelength tunability, high power and multi-wavelength property have been reported in recent years, nevertheless, the linewidth parameter of laser spectrum, which is related to the inherent temporal property of a laser, its manipulation has not been reported so far but merits detailed investigation for the sake of application potential in sensing and communication systems based on RFLs. In this presentation, we demonstrate a wavelength-tunable, linewidth-adjustable high power RFL with linearly polarized output for the first time to our best knowledge, in which the central wavelength and the linewidth of the spectrum can be tuned independently by inserting a bandwidth-adjustable tunable optical filter (BA-TOF). This laser provides a flexible tool for scenes where the temporal coherence property accounts, for example, coherent sensing/communication and nonlinear frequency conversion. In the proof-of-principle experiment, the central wavelength can be continuously tuned from 1095 to 1115 nm, while the maximal tuning range of the full width at half-maximum (FWHM) linewidth is from ~0.6 to more than 2 nm. Moreover, the output power of 1102.5–1112.5 nm reaches ~23 W with polarization extinction ratio (PER) value > 20 dB.

## Results

### Operation without BA-TOF

**Figure 1:**
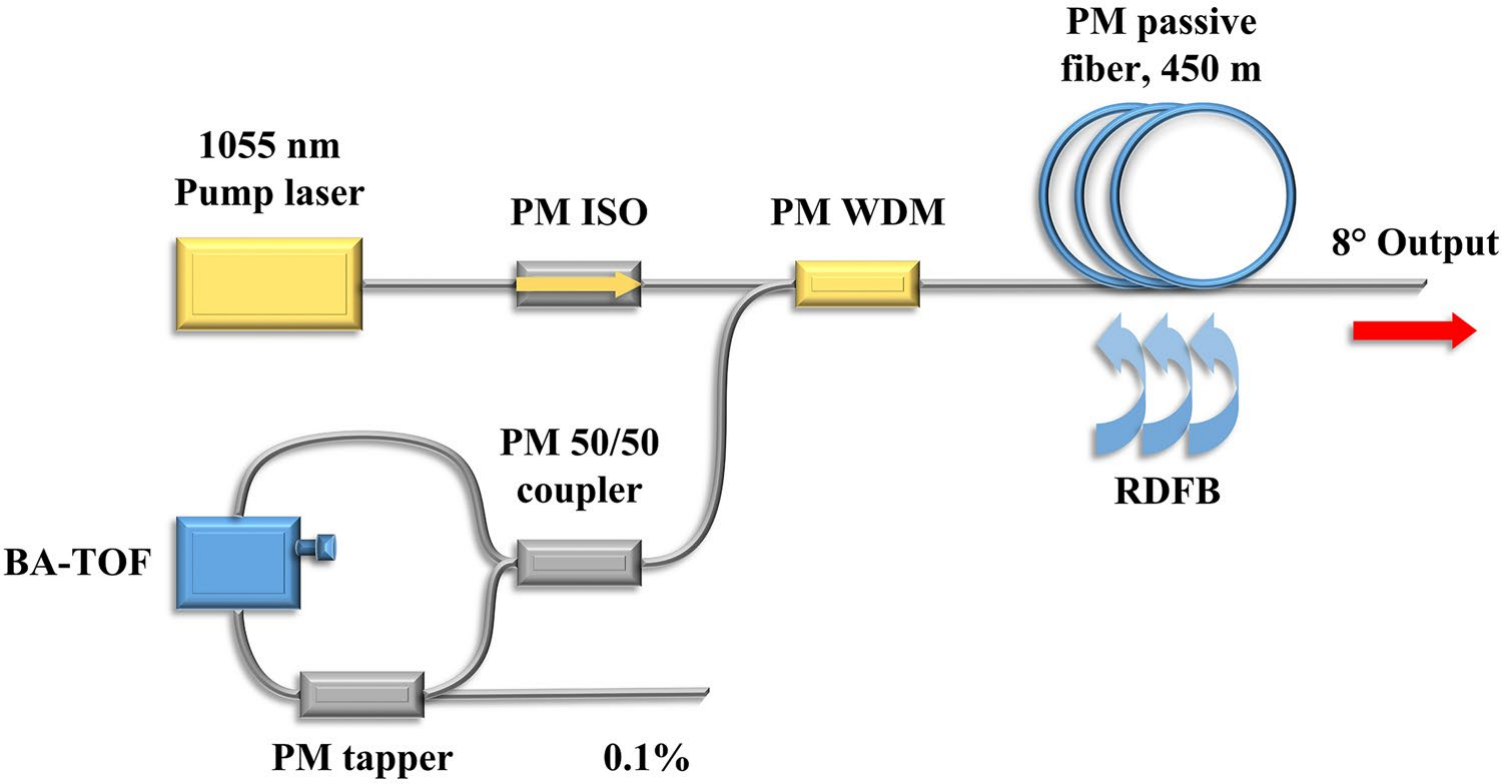
Schematic diagram of the experimental setup. PM ISO: Polarization maintaining isolator, PM WDM: Polarization maintaining wavelength division multiplexer, BA-TOF: Bandwidth-adjustable tunable optical filter, RDFB: Random distributed feedback.

The experimental setup is schematically shown in Fig. 1. It includes a linearly polarized fiber laser centered at 1055 nm as the pump source, followed by a polarization maintaining isolator (PM ISO). A half-opened cavity structure which consists of a fiber loop mirror (FLM) and a piece of 450-m-long PM passive fiber is utilized to decrease the threshold of random lasing. A BA-TOF is placed into the FLM to obtain wavelength-tunable and linewidth-adjustable random emission. In order to monitor the operation of the BA-TOF, a PM tapper with coupling ratio of 1/999 is also placed into the FLM. In addition, all the end facets are cleaved at an angle of 8° to suppress the unexpected backward reflection.

**Figure 2:**
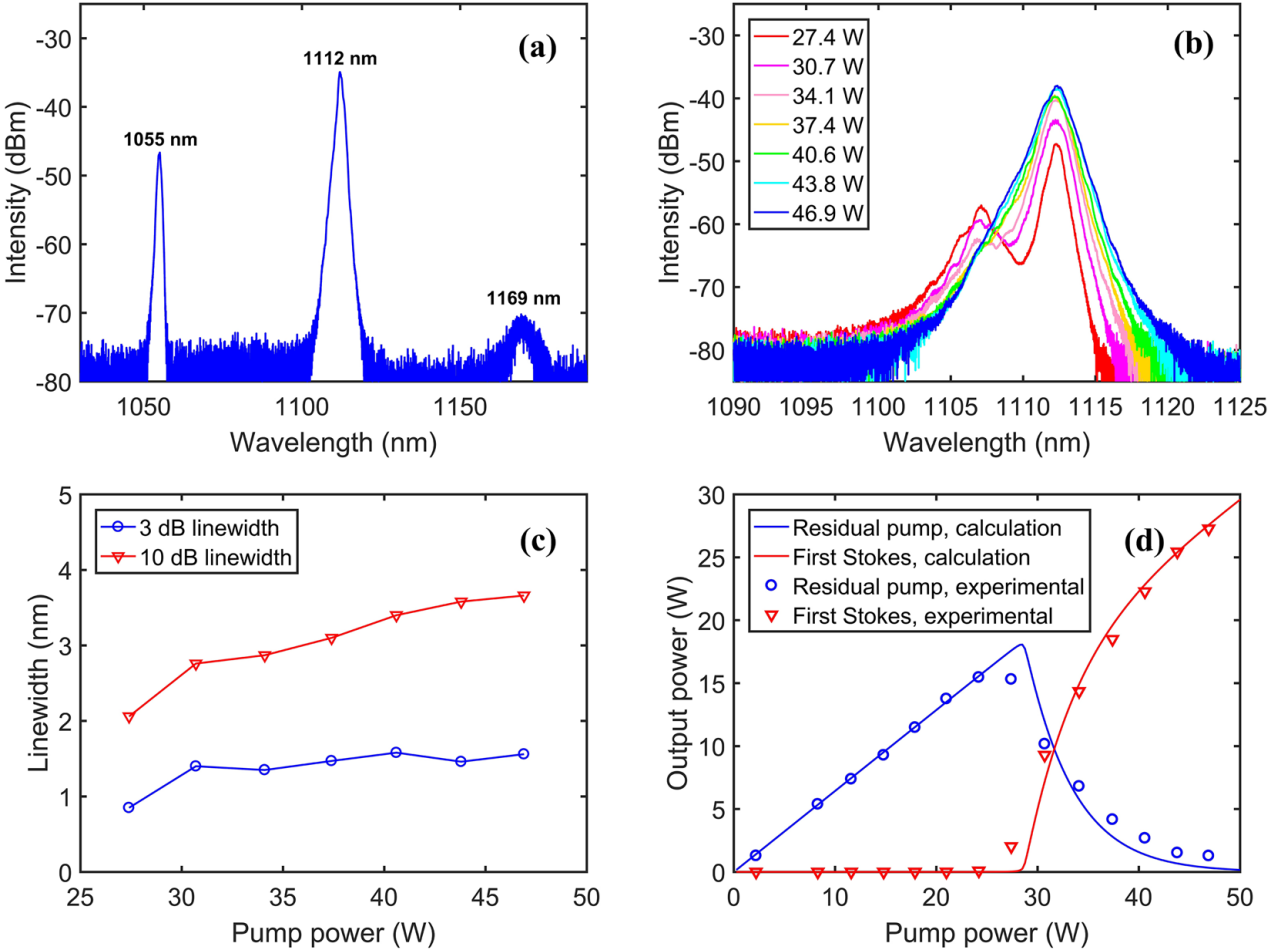
(**a**) Spectrum at the maximal pump power of 46.9 W. (**b**) Spectra of the first order Stokes at different pump powers. (**c**) FWHM linewidth and 10 dB linewidth evolutions. (**d**) Output power as a function of the injected pump power.

As a comparison experiment, we first studied the linearly polarized RFL operating without the BA-TOF. Figure 2(a) shows the output spectrum with maximal pump power of 46.9 W, and it’s seen that the spectrum is stable and smooth, which consists of three components: the residual 1055 nm pump radiation, the first order Stokes centered at 1112.3 nm and the second order Stokes centered at 1169 nm. The intensities of the residual 1055 nm pump radiation and the second order Stokes are measured to be 12 dB and 35 dB lower than the first order Stokes, respectively, indicating the high power purity up to 95% (calculated through numerical integration based on the spectral data). We also measured the spectral evolution of first order Stokes at different pump powers, as presented in Fig. 2(b). At lower pump power (<31 W), it’s found that the spectrum consists of two subcomponents at 1107.1 nm and 1112.3 nm, probably because the Raman gain profile in silica fibers has two nearly equal peaks shifted by ~13.2 THz and ~14.6 THz relative to the pump wave^10^. Whereas with the increment of pump power, the second subcomponent gradually takes a dominant role and eventually forms the spectrum with a single-peak structure. This spectral feature has been well explained in ref.^29^ by considering power transfer between adjacent subcomponent.

Figure 2(c) shows the linewidth evolution as a function of the pump power. The spectra rapidly broaden when the injected pump power is relatively low (<30.7 W), and the FWHM linewidth varies from ~0.8 nm to ~1.4 nm while the 10 dB linewidth increases from ~2 nm to ~2.8 nm correspondingly. When the pump power is increased further, the FWHM linewidth saturates at 1.5–1.6 nm level, whereas the 10 dB linewidth keeps near-linearly increasing to ~3.7 nm. The spectral broadening could be explained by the nonlinear effects such as cross-phase modulation (XPM) and self-phase modulation (SPM)^30^. By measuring the total output power and doing integration based on the spectral data, we can get the power of the residual pump and the first order Stokes. Figure 2(d) shows the output powers dependence on the pump power, it’s seen that the lasing threshold of the first order Stokes is about 27 W, below which only the residual pump radiation is present. Above the lasing threshold, the output power of the first order Stokes grows rapidly, which reaches 27.3 W with full pump power of 46.9 W. The power ratio of the first order Stokes to the injected pump radiation, or the so-called optical-optical conversion efficiency, is calculated to be ~57%. In addition, the simulated output powers (presented by a blue line and a red line) through a power balance model^31,32^ coincide well with the experimental output powers (presented by blue circles and upside-down red triangles).

### Wavelength Tunability

**Figure 3:**
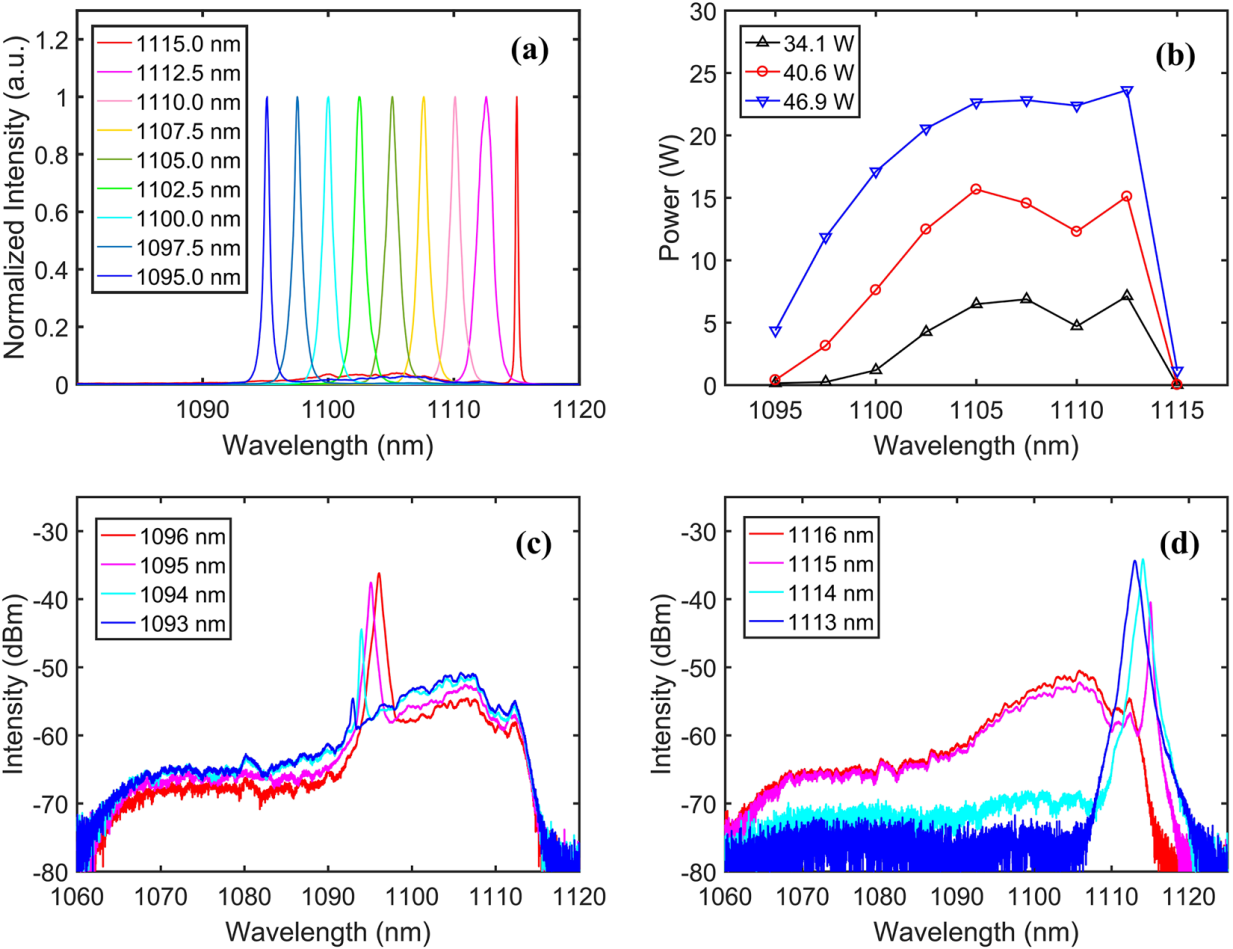
(**a**) Normalized tunable output spectra. (**b**) Output power of the first order Stokes as a function of wavelength with the pump power of 34.1 W, 40.6 W and 46.9 W. (**c**) Spectra near 1095 nm and (**d**) 1115 nm.

Inserting the BA-TOF and fixing its passband at the minimum value, we first explored the wavelength tunability of the RFL. The normalized output spectra are shown in Fig. 3(a), which are measured from 1095 nm to 1115 nm with a step of 2.5 nm, spanning a spectral range of 20 nm. Figure 3(b) shows the output power of the first order Stokes dependence on the operating wavelength with the pump power of 34.1 W, 40.6 W and 46.9 W. An apparent similarity between the power variation curve and the Raman gain profile can be observed, where the output powers reach the highest value at ~1107.5 nm and ~1112.5 nm, corresponding to the two peaks of the Raman gain with frequency shifts of ~13.2 THz and ~14.6 THz. Furthermore, the maximal output powers of 1102.5–1112.5 nm reach more than 20 W with power fluctuation less than 1 dB. But at shorter and longer wavelengths, the Raman gain decreases significantly, which causes the obvious decrease of optical-signal-to-noise ratio (OSNR) and thus leads to the clear reduction of output powers. As shown in Fig. 3(c), when operating at 1096 nm and 1095 nm, the signal lasing is much more powerful than the spontaneous Raman emission, and the OSNRs are measured to be ~21 dB and ~15 dB respectively. Whereas at 1094 nm, the OSNR quickly reduces to ~7 dB. Further decreasing the wavelength to 1093 nm, the signal lasing almost fully degrades and only the spontaneous Raman emission is present in the output spectrum. A similar case occurs near 1115 nm, as shown in Fig. 3(d), the OSNR varies from 34 dB to 12 dB when tuning the central wavelength from 1114 nm to 1115 nm. Similarly, the signal lasing is difficult to radiate when further adjusting the wavelength to 1116 nm. As a result, due to the available bandwidth of the Raman gain, the wavelength tuning range is confined to ~20 nm, which is a typical value in tunable RFLs with only Raman gain^22–24^. By optimizing the passband of the BA-TOF and the length of the passive fiber, perhaps the wavelength tuning range can be further expanded.

### Linewidth Tunability

**Figure 4:**
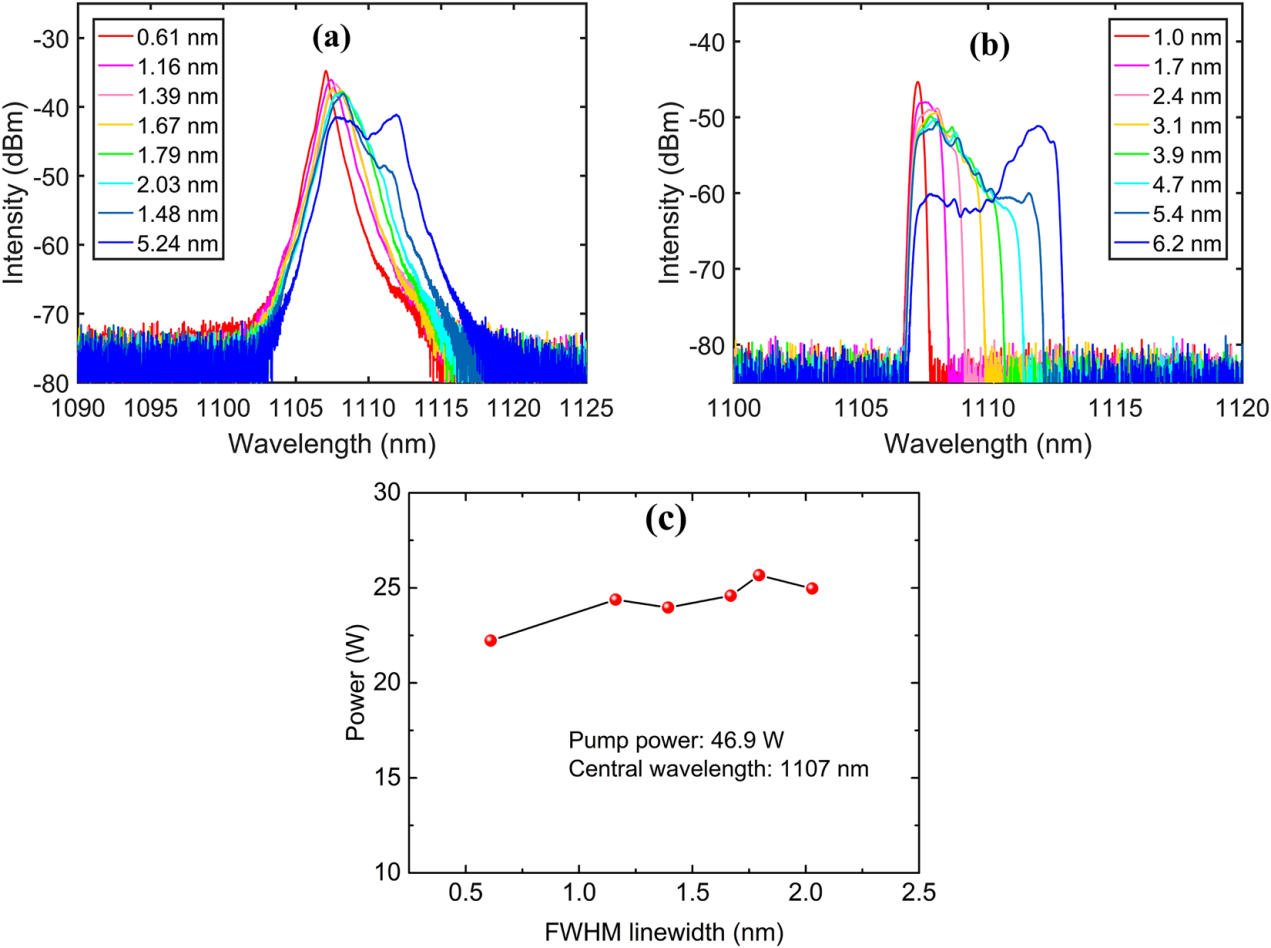
(**a**) Linewidth-adjustable spectra of the output beam and (**b**) the 0.1% sample light. (**c**) Output power as a function of the FWHM linewidth.

On the other hand, fixing the central wavelength, we analyzed the linewidth tunability of the RFL. Taking the case of operating at 1107 nm, the linewidth-adjustable spectra of the output beam and the 0.1% sample light in the FLM are depicted in Fig. 4(a) and (b) respectively (measured at full pump power). It should be noted that the legend of Fig. 4(a) presents the FWHM linewidths while the legend of Fig. 4(b) stands for the passband of the BA-TOF. The output spectrum shows a clear peak with relatively long tails, while the spectrum in the FLM presents a flatter top with sharp edges. Via increasing the passband of the BA-TOF, the output spectra can gradually broaden, where the FWHM linewidths can be continuously tuned from ~0.61 nm to ~2.03 nm (the corresponding 10 dB linewidth varies from ~1.97 nm to ~4.02 nm). An interesting phenomenon can be observed when further increasing the passband of the BA-TOF, that is the abrupt change of the FWHM linewidth. When adjusting the passband to 5.4 nm, the FWHM linewidth narrows to ~1.48 nm. However, when the passband is increased to 6.2 nm, the FWHM linewidth immediately broadens to ~5.24 nm.

The reason could be attributed to the working principle of the BA-TOF, whose passband is increased via broadening the aperture inside to let the longer wavelength pass through, and the linewidth-adjustable spectra in the FLM also testify this working principle (see Fig. 4(b)). Thus, due to the high Raman gain at frequency shift of ~14.6 THz, the subcomponent near 1112 nm will start to radiate immediately as long as this wavelength is covered by the passband of the BA-TOF. As a result, the subcomponent of ~1107 nm and the subcomponent of ~1112 nm are simultaneously present in the output spectrum, thus leading to the abrupt change of the FWHM linewidth. In fact, the concept of FWHM linewidth is not universally applicable for single-peak spectrum and multi-peak spectrum. Also due to the working principle of the BA-TOF, the central wavelength is moving little by little away from 1107 nm when adjusting the linewidth. Figure 4(c) shows the output power of the random emission while tuning the linewidth (measured at 1107 nm with 46.9 W pump). The laser output power reaches ~23 W and keeps almost constant with a variation of ~8% in the tuning range 0.6–2.0 nm.

### Simultaneous Manipulation of Wavelength and linewidth

**Figure 5:**
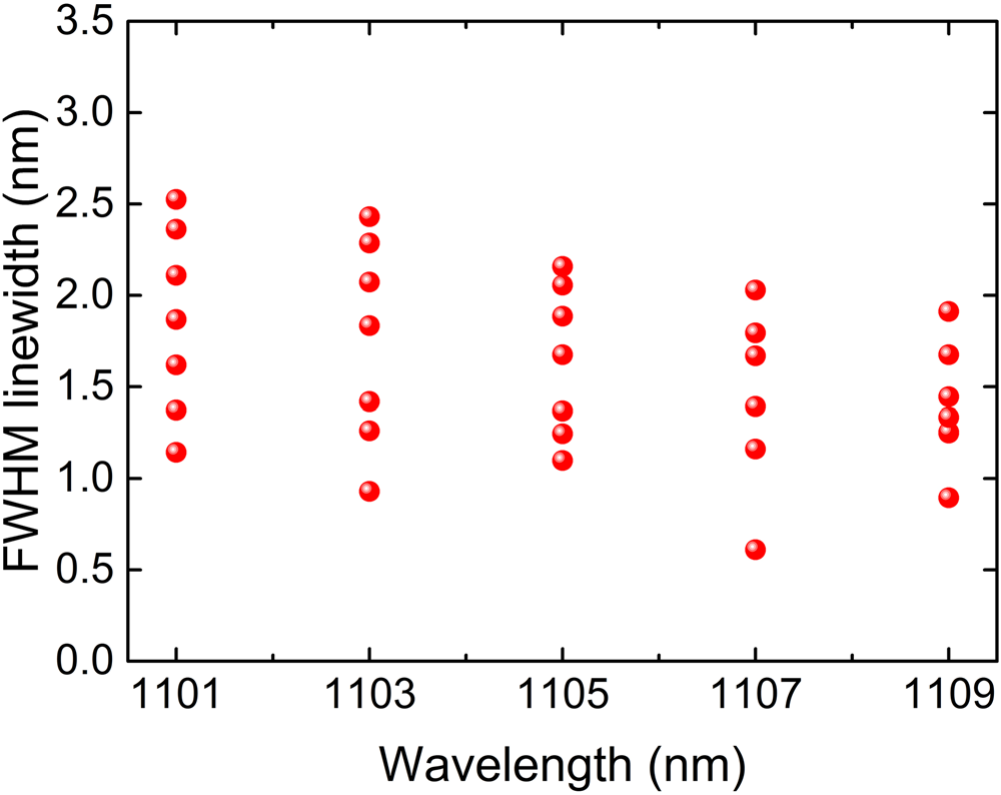
Tunable linewidths at different central wavelength.

Furthermore, we studied the linewidth tunability at different wavelength. Increasing the passband of the BA-TOF with a step of ~0.7 nm, we measured the FWHM linewidths of the spectra with central wavelength varying from 1101 nm to 1109 nm. As shown in Fig. 5, the presented configuration could provide a continuous tuning range of ~1.5 nm for the linewidth-adjustable random emission. At a certain central wavelength, the broadest spectrum shows a FWHM linewidth 2–3 times that of the narrowest one. The linewidth tuning range may be limited by the width and the flatness of the convolution between the Raman gain profile and the spectrum of the pump radiation.

Finally, the PER value of the linearly polarized RFL is analyzed briefly, which is measured by a spatial structure constructed as follows. At the output end, a collimator is used to avoid the divergence of the output beam, after which a dichroic mirror (DM) is employed to extract the first order Stokes from the remaining 1055 nm pump radiation. The polarization direction of the first order Stokes should be adjusted by a half-wave plate, so that the polarization beam splitter (PBS) can split the input beam into two components with maximal power difference, thus dividing the two beams with orthogonal polarization direction. The PER value of the output laser is defined as *PER* = *10 log(P*_*max*_*/P*_*min*_), where *P*_*max*_ and *P*_*min*_ represent the maximal and the minimal power of the beams split by the PBS when rotating the half-wave plate. As a result, with the pump power grows and well above the threshold, we found that the PER value keeps nearly the same, slightly varying between 21 and 22 dB.

## Discussion

Note that the wavelength tuning range is confined to ~20 nm, owing to the available bandwidth of the Raman gain, which is only a few THz although it nominally covers a range over 40 THz^33^. In our case, the practicable gain coverage is measured to be 10.5–15.2 THz (about 4.7 THz), which is a typical value in tunable RFLs with only Raman gain. For example, the available bandwidths of the Raman gain are 4.3 THz in ref.^22^, 2.6 THz in ref.^23^ and 1.4 THz in ref.^24^, respectively. And in fact, if counting only the wavelength with high output power and high efficiency, the calculated available gain coverage will be much narrower. In this experiment, the wavelength with output power higher than 20 W ranges from ~1102 to ~1113 nm, hence the corresponding available gain coverage is calculated to be 12.1–14.5 THz (about 2.4 THz). Through optimizing the length of the PM passive fiber (may not be the optimal length for highest output power), reducing losses in the experimental setup or combining different gain mechanisms, the wavelength tuning range is possible to be further expanded. On the other hand, the maximal tuning range of the FWHM linewidth is from ~0.6 to ~2 nm at the full pump power. The linewidth tuning range may be limited by the width and the flatness of the convolution between the Raman gain profile and the spectrum of the pump radiation. Therefore, a pump source with broadband spectrum could be a good candidate to further widen the linewidth tuning range. Moreover, by ingeniously designing the reflectivity spectrum of a fiber Bragg grating (FBG) to compensate the unevenness of the Raman gain, we are more likely to obtain a broader linewidth tuning range with customized spectrum profile.

In summary, a high power linearly polarized RFL with flexible spectral manipulation property is reported in this manuscript, especially the linewidth tunability, is explored for the first time as far as we know. The presented high power linearly polarized RFL has a typical half-opened configuration and employs a BA-TOF to obtain wavelength-tunable and linewidth-adjustable random emission at the same time. As a result, the central wavelength can be continuously tuned from 1095 to 1115 nm, while the FWHM linewidth has a maximal tuning range from ~0.6 to more than 2 nm. Moreover, the output power of 1102.5–1112.5 nm reaches ~23 W with PER value > 20 dB. This laser may provide a flexible tool for scenes where the temporal coherence property accounts, such as coherent sensing/communication and nonlinear frequency conversion. The next step of our research will pay attention to further expand the tuning ranges and explore the applications in tunable optical parametric oscillator (OPO) and visible light generation. Meanwhile, combining with the broadband saturable absorption property of two-dimension materials to realize the temporal and spectral manipulation of RFLs may bring great interest for practical application and scientific research^34–36^.

## Methods

The pumping is provided by a linearly polarized all-fiber MOPA source centered at 1055 nm, which consists of a continuous wave (CW) ring-cavity seed and a PM Yb-doped fiber amplifier. The power of the radiation emitted from the seed is measured to be ~1.04 W, and after passing through the PM Yb-doped fiber amplifier, the pump laser can generate linearly polarized emission with an output power up to 46.9 W and FWHM linewidth <1.2 nm. Then the linearly polarized pump radiation is injected into a piece of 450-m-long PM passive fiber (PM Ge-doped fiber with a core/inner cladding diameter of 10/125 μm and numerical aperture of 0.07) via the 1060 nm port of a high power PM wavelength division multiplexer (PM 1060/1110 nm WDM with operating range ± 10 nm), the 1110 nm port of the PM WDM is spliced to a PM coupler with coupling ratio of 50/50, which forms a fiber loop mirror (FLM) by splicing its output ports. Thus, a linearly polarized RFL with a half-opened cavity is constructed, in which the feedback is provided both by the broadband reflective FLM and the random distributed Rayleigh scattering in the passive fiber. To obtain wavelength-tunable and linewidth-adjustable random emission, a BA-TOF with 1-m-long pigtailed fiber is placed into the FLM, which is built based on free-space optical Fourier transformation combing with diffraction gratings to provide an access of selecting spatially desired spectral ingredients of input light and makes it possible to tune the central wavelength and the linewidth independently. The output powers and the spectra are measured by a power meter and an optical spectrum analyzer (Yokogawa AQ6370D), respectively.
